# Loquat-tea intercropping enhances rhizosphere microbial diversity and functional profiles in tea soil ecosystems

**DOI:** 10.3389/fmicb.2025.1651997

**Published:** 2025-08-07

**Authors:** Chengyi Zou, Tianfei Dai, Zhonghua Liang, Mingyan Li, Lina Meng, Xianming Zhao, Nianzhen Li, Qin Wei, Mohamed A. Abd Elhamid, Amr M. Atif, Salma A. Soaud, Mengling Wen, Kuan Yan, Ahmed H. El-Sappah

**Affiliations:** ^1^Faculty of Agriculture, Forestry and Food Engineering, Yibin University, Yibin, China; ^2^Sichuan Green Food Development Center, Chengdu, China; ^3^Xingwen Sanhe Landscaping Co., Ltd., Yibin, China; ^4^Department of Genetics, Faculty of Agriculture, Zagazig University, Zagazig, Egypt; ^5^Department of Microbiology, Faculty of Agriculture, Zagazig University, Zagazig, Egypt

**Keywords:** intercropping, tea rhizosphere, metagenomics, microbial diversity, functional genes

## Abstract

**Introduction:**

Intercropping systems can significantly influence soil microbial communities, affecting plant health and soil nutrient cycling, which has better economic and ecological benefits than monoculture of tea.

**Methods:**

This study investigated the impact of loquat-tea intercropping on the microbial community structure and functional gene composition in the rhizosphere soil of tea (*Camellia sinensis*). Using metagenomic sequencing, we analyzed rhizosphere soils from loquat-tea intercropping (PP_CS), tea monoculture (CS), and loquat monoculture (PP).

**Results and discussion:**

A total of 161 phyla, 269 classes, 485 orders, 1,000 families, 3,838 genera, and 27,624 species were annotated across samples. Dominant phyla included *Actinobacteria*, *Proteobacteria*, *Acidobacteria*, and *Chloroflexi*. The genera *Bradyrhizobium* (4.20%) and *Trebonia* (3.78%) were notably enriched in the intercropping system. The analysis of community differences showed that unclassified_c_acidobacteria was in pp_cs group, demonstrating the highest LDA score (4.4 score). Functional annotation via the Kyoto Encyclopedia of Genes and Genomes (KEGG) revealed that metabolic pathways were predominant across all treatments, with 36,111,608 reads assigned to metabolism. The comparative analysis at KEGG level 3 revealed that *Metabolic pathways* 289 constituted the most abundantly annotated functional category across all three groups. Redundancy analysis (RDA) showed strong correlations between key microbial genera (*Trebonia*, *Bradyrhizobium*) and soil properties, including organic matter (OM), alkali hydrolyzed nitrogen (AN), available phosphorus (AP), and available potassium (AK). These findings suggest that loquat-tea intercropping promotes microbial diversity and enhances functional potential, improving soil health and nutrient availability in tea cultivation systems.

## 1 Introduction

*Camellia sinensis* is a healthy beverage crop with a unique aroma and taste, as well as anti-cancer, anti-aging, and other medicinal and health benefits, which people love (Wang R. et al., 2018; Wang Z. J. et al., 2018). After long-term adaptation and evolution, tea plants have formed ecological habits that can tolerate shade, temperature, and humidity, like diffuse light and acid soil, and are widely planted in Southwest China (Yan et al., 2017). The economic benefits of tea gardens, as well as the improvement of tea quality and safety, are consistently the primary goals of the tea industry (Xie et al., 2021). With the continuous expansion of tea planting area, water and soil loss, soil fertility reduction, and large-scale growth of weeds in tea plantations pose a serious threat to the yield and quality of tea. The use of herbicides, pesticides, and other chemical pesticides not only caused severe damage to the ecological environment of tea gardens but also posed a threat to human health. Therefore, implementing reasonable and scientific farming technology to improve the soil fertility of tea gardens, as well as constructing ecological tea gardens, are important measures to ensure the synchronous improvement of both tea yield and quality. The results show that intercropping can not only significantly improve crop yield and stability but also fully utilize the resources of light, heat, water, and nutrients, thereby strengthening the service function of the farmland ecosystem (Li, 2016; Liu et al., 2016). Intercropping in tea gardens can not only prevent soil erosion and stabilize the tea garden ecosystem but also improve soil fertility. Intercropping can effectively increase the species diversity of tea gardens and maximize the utilization of tea garden space and resources, and thus have an impact on the growth and development of tea plants, the quality characteristics of tea and the yield and other physiological ecology, making it an important production mode in modern ecological agriculture (Duchene et al., 2017). Yao et al. (2016) found that the levels of free amino acids, water extracts, caffeine, and water-soluble carbohydrates of tea in pear tea intercropping tea plantations were significantly higher than those in monoculture tea plantations. Chen et al. (2011) found in their research on pear tea intercropping, that intercropping can enhance nitrogen metabolism and weaken the respiratory metabolic pathway of tea plants by enhancing diffuse radiation illumination and changing light quality, to improve the level of amino acids in tea, reduce the ratio of phenol to ammonia in tea, and make green tea taste fresh. Studies have found that intercropping *Eriobotrya japonica* in tea gardens can improve the content of soil nutrients, improve the soil properties of tea gardens, and then improve the yield and nutritional quality of tea (Liu et al., 2017). Therefore, reasonable and diversified planting in tea gardens is of great significance for improving the soil ecological environment in tea gardens and promoting the sustainable development of efficient and high-quality tea production.

Soil microorganisms are the core of the soil ecosystem and play a crucial role in controlling and mitigating soil pollution, maintaining soil nutrient balance, and promoting the soil biogeochemical cycle (van der Heijden et al., 2008; Fu et al., 2020; Wang R. et al., 2018; Wang Z. J. et al., 2018). Increasingly, studies have shown that the relationship between soil nutrient factors and soil microorganisms can provide a deeper understanding of the ecological processes and mechanisms underlying soil microorganisms’ role in promoting normal plant growth (Lange et al., 2014). Further study on the interaction mechanism between microorganisms and soil nutrients, particularly the functional role of microorganisms, is of great significance for a deeper understanding of nutrient absorption and plant growth and development.

Previous studies on soil microorganisms mainly focused on the distribution characteristics of microbial community structure and its influencing factors. For example, studies have found that *Proteobacteria*, *Actinomycetes*, *Bacteroides*, and *Firmicutes* are the dominant bacterial groups in desert soil (Li T. et al., 2018; Li M. S. et al., 2018). Wang R. et al. (2018) and Wang Z. J. et al. (2018) demonstrated that the diversity of the soil bacterial community increased significantly over time in soil natural restoration years, and the community structure also underwent significant changes. Soil microorganisms can not only promote plant growth by producing metabolites but also inhibit the growth of pathogenic microorganisms by inducing plants to play an antagonistic role, thereby maintaining the dynamic balance of the soil ecosystem. Studies have found that *Trichoderma harzianum* can effectively inhibit black scurf of potatoes (Abdul Rauf et al., 2015). The decomposition effect of soil microorganisms can accelerate the absorption of nutrients by plants, at the same time, disintegrate soil masses, dredge soil pores, promote gas exchange in the soil, and then be conducive to the respiration of plant roots. In the intercropping system, the interspecific competition and promotion effect of different plants will often enrich the diversity of microbial communities in the soil, and the increase in the number of microorganisms will also reverse the regulation of plant growth. Under the appropriate intercropping mode, the two can achieve the effect of mutual benefit and common promotion. Therefore, the change of soil microbial quantity is an index that can best reflect the soil condition, and is also related to the effect of intercropping mode on soil activity.

A large number of studies have shown that intercropping can increase the number of soil microorganisms, change the structure of the soil microbial community, and control plant diseases (Chen et al., 2019). For example, the number of bacteria, actinomycetes, and fungi increased under the intercropping mode of tea and soybean, while the microbial biomass and diversity index were also improved, so as to repair the soil with tea wilt disease (Qin et al., 2019). Due to the high functional redundancy of microorganisms, accurately predicting their functional characteristics is challenging. In contrast, functional genes can better predict the response of microbial communities to habitat changes. In particular, the advancement of metagenome sequencing has significantly enhanced the ability to characterize the environmental metagenome (Fierer et al., 2012; Burke et al., 2011; Luo et al., 2023). Metagenomic sequencing directly extracts total DNA from soil samples. Compared with amplicon sequencing, it can not only reflect the structural changes of microbial communities more comprehensively, accurately, and truly, but also provide information on microbial functions, further improving the understanding of microbial communities and their roles in the environment (Wu et al., 2017; Ma et al., 2015).

Plant planting pattern is one of the key factors affecting soil quality, and reasonable space-time collocation between plants can effectively improve soil quality. Reasonable intercropping can significantly improve the photosynthesis of plants, inhibit the breeding of weeds and pests, and improve the soil microecological environment. Compared with tea monoculture, tea fruit intercropping not only brings more economic benefits to tea farmers but also lays an ecological foundation for the development of organic tea gardens in the region (Qin et al., 2017). The different patterns of tea-fruit intercropping will produce different ecological effects, and also have different effects on the growth, quality, and yield of tea plants. The crown width and root size of tea trees are small, and the density is low. There are a large number of empty niches in the tea garden land, which provides the possibility of intercropping with fruit trees.

At present, most researches focus on the diversity of soil microbial community in the field crop intercropping (Ma et al., 2016), while the research on the structure of soil microbial community in the tea tree and fruit tree intercropping is relatively lack, especially the research on the comprehensive understanding of the functional diversity of rhizosphere microorganisms in the intercropping soil by using metagenomics is still blank. Therefore, in this study, metagenomic sequencing technology was employed to analyze the rhizosphere soil microbiota of tea-loquat intercropping systems, exploring the interactions between rhizosphere microbial community structures, gene functions, and soil nutrient influencing factors, which provided a theoretical basis for revealing the interaction mechanism between rhizosphere microorganisms and soil nutrients under tea and fruit intercropping mode, and also provided a scientific basis for improving the soil environment of tea garden, improving the yield and quality of tea, and further promoting the efficient and safe production of tea.

## 2 Materials and methods

### 2.1 Overview of test site

The test site is Yibin tea planting base (E 104°72′, N 28°93′) in Sichuan Province. The climate there is a humid subtropical monsoon climate with four distinct seasons, rainy and hot seasons, sufficient sunlight, long frost free period, mild and humid temperature, annual average temperature of 17.9°C, rainfall of 1,123 mL, sunshine of 2,278 h, frost free period of 344 days, which is conducive to the growth of tea, loquat, plum and other crops. The soil is acid red soil, which contains alkali hydrolyzable nitrogen 57.44 mg/kg, available phosphorus 14.78 mg/kg, available potassium 144.74 mg/kg, organic matter 1.17 g/kg, and pH 5.34.

Both loquat and tea trees have been planted since 2017. The row spacing of monoculture tea trees is 40 cm × 50 cm, and that of monoculture loquat trees is 440 cm × 440 cm. In the intercropping tea garden, one loquat tree is planted for every three tea trees. The row spacing of tea trees is 180 cm × 70 cm, and that of loquat trees is 180 cm × 300 cm. According to the local actual production, the tea garden mainly applies nitrogen, phosphorus, potassium fertilizers (the application amount is 450, 60, 90 kg/m^2^ respectively) and some local conventional organic fertilizers. During the whole experiment period, all the tea garden experimental plots adopted the same cultivation management mode.

### 2.2 Sample collection

To investigate the soil characteristics associated with different cropping systems, we established three distinct soil types: loquat monoculture (PP), tea monoculture (CS), and intercropping of loquat and tea (PP_CS). For each soil type, three sampling plots, each measuring 20 m × 20 m, were delineated. Within each plot, five sampling points were selected following the five-point sampling method. Using a trowel disinfected with 75% ethanol, we excavated the main roots and lateral rootlets from a depth of 10–30 cm, within a radial distance of 30–50 cm from the plant trunk. The rhizosphere soil, defined as the soil closely adhering (0–4 mm) to the root surfaces, was carefully collected using a sterile brush, with an approximate total weight of 5–10 g per sample. For each replicate, samples from five plants were pooled and homogenized, then transferred into 50-mL centrifuge tubes. These samples were subsequently stored at −80°C and transported to the laboratory for DNA extraction. The extracted DNA was then sent to Majorbio Bio-Pharm Technology Co., Ltd. (Shanghai, China) for metagenomic sequencing.

### 2.3 Microbial DNA extraction and metagenome sequencing

Microbial DNA extraction was conducted using the E.Z.N.A.^®^ reagent kit (OMEGA^®^, United States), following the specific instructions provided in the kit. After DNA extraction was completed, the concentration and purity of the DNA were assessed, and its integrity was verified using 1% agarose gel electrophoresis. Each treatment group consisted of three biological replicates (*n* = 3), and equal amounts of DNA from each replicate were used for sequencing. A certain number of qualified DNA samples were sent to the Illumina HiSeq4000 platform (Illumina Inc., San Diego, CA, United States) for terminal sequencing. To monitor for potential contamination, extraction blanks (no-template controls) were included during DNA extraction and sequencing library preparation and were processed alongside the samples. No significant DNA amplification or sequencing reads were detected in these negative controls. Raw sequencing reads were processed using fastp^1^ to trim adapter sequences from both 3′ and 5′ ends. Low-quality reads were filtered through the following criteria: removal of reads with post-trimming lengths <50 bp, average quality scores <20, or ambiguous bases (N). High-quality paired-end and single-end reads were retained for subsequent metagenomic analysis. Sequences with varying sequencing depths were assembled *de novo* using MEGAHIT.^2^ Contigs of 300 bp or longer were selected as the final assembly outputs and subjected to downstream gene prediction and annotation. Open reading frames (ORFs) within contigs were predicted using MetaGene.^3^ Predicted genes with nucleotide sequences ≥100 bp were translated into amino acid sequences, generating a statistical table of gene prediction results for each sample.

All predicted gene sequences across samples underwent clustering analysis via CD-HIT,^4^ employing thresholds of ≥95% sequence identity and ≥90% coverage. A non-redundant gene set was constructed by selecting the most extended sequence as the representative for each cluster. Quality-controlled reads were aligned against the non-redundant gene set using SOAPaligner^5^ with a sequence identity of ≥95%, enabling the quantification of gene abundance in each sample.

For functional annotation, the non-redundant gene set was aligned against the NCBI-NR and Kyoto Encyclopedia of Genes and Genomes (KEGG) databases using BLASTP^6^ with an *E*-value threshold of 1 × 10^−5^. Taxonomic and functional annotations were derived from these alignments. Species- or function-specific abundance profiles were calculated through the summation of gene abundances corresponding to each taxonomic or functional category.

### 2.4 Determination of soil physical and chemical properties

The physical and chemical properties of soil were determined by the method of Wang et al. (2022). Soil pH was measured using a calibrated pH meter. Soil organic matter (OM) was determined by the potassium dichromate-sulfuric acid oxidation method. Total nitrogen (TN) content was quantified via the Kjeldahl distillation method. Available phosphorus (AP) was analyzed using the molybdenum-antimony ascorbic acid colorimetric method, while available potassium (AK) was measured by atomic absorption spectrophotometry.

### 2.5 Data processing and statistical analysis

Taxonomic annotation was performed using the non-redundant (NR) reference database,^7^ with species abundance calculated by summing gene abundances corresponding to each taxon. Hierarchical abundance profiling was subsequently conducted across eight taxonomic ranks: Domain, Kingdom, Phylum, Class, Order, Family, Genus, and Species. Taxon-specific abundance tables were generated at each taxonomic level to characterize microbial community composition across all samples systematically.

Analytical procedures, including microbial community composition profiling, community heatmap, diversity assessment, and Linear Discriminant Analysis Effect Size (LEfSe) for differential taxa identification, were conducted using RStudio (v4.3.2) (Lu et al., 2016; Zhou et al., 2017). Redundancy analysis (RDA) was implemented via the vegan package (v2.6-4) to elucidate relationships between soil physicochemical parameters and functional microbial community composition.

The non-redundant gene set was aligned against the KEGG GENES database using DIAMOND^8^ with BLASTP parameters (*E*-value ≤1 × 10^−5^). Functional abundance was calculated by aggregating gene abundances corresponding to each KEGG Orthology (KO) and Pathway. This enabled systematic profiling of functional potential across samples at both KO and pathway levels.

Statistical significance tests and correlation analyses of soil physicochemical properties were performed using SPSS 21.0 (SPSS Inc., Chicago, IL, United States). Data visualization was conducted using Excel 2021 (Microsoft Corp.). A comparative analysis of microbial functional gene relative abundance was performed between monocropping and intercropping systems using STAMP software (v2.1.3). All data are presented as mean values with standard deviations (SD) and analyzed using one-way analysis of variance (ANOVA). A Duncan multiple-comparison test was applied to detect variations among means of all samples at a *p*-value *<*0.05 level of significance.

## 3 Result

### 3.1 Microbial community composition

The study encompassed nine biological samples, generating 468,244,322 raw reads through initial sequencing. Following stringent quality control (Q30 threshold, adapter trimming), 460,863,082 high-quality clean reads were retained, achieving sample-wise coverage ≥98% across all replicates. This robust sequencing depth (average of ~51.2 million reads per sample) confirms adequate resolution for downstream metagenomic analyses, including the detection of rare taxa and functional profiling.

**Figure 1:**
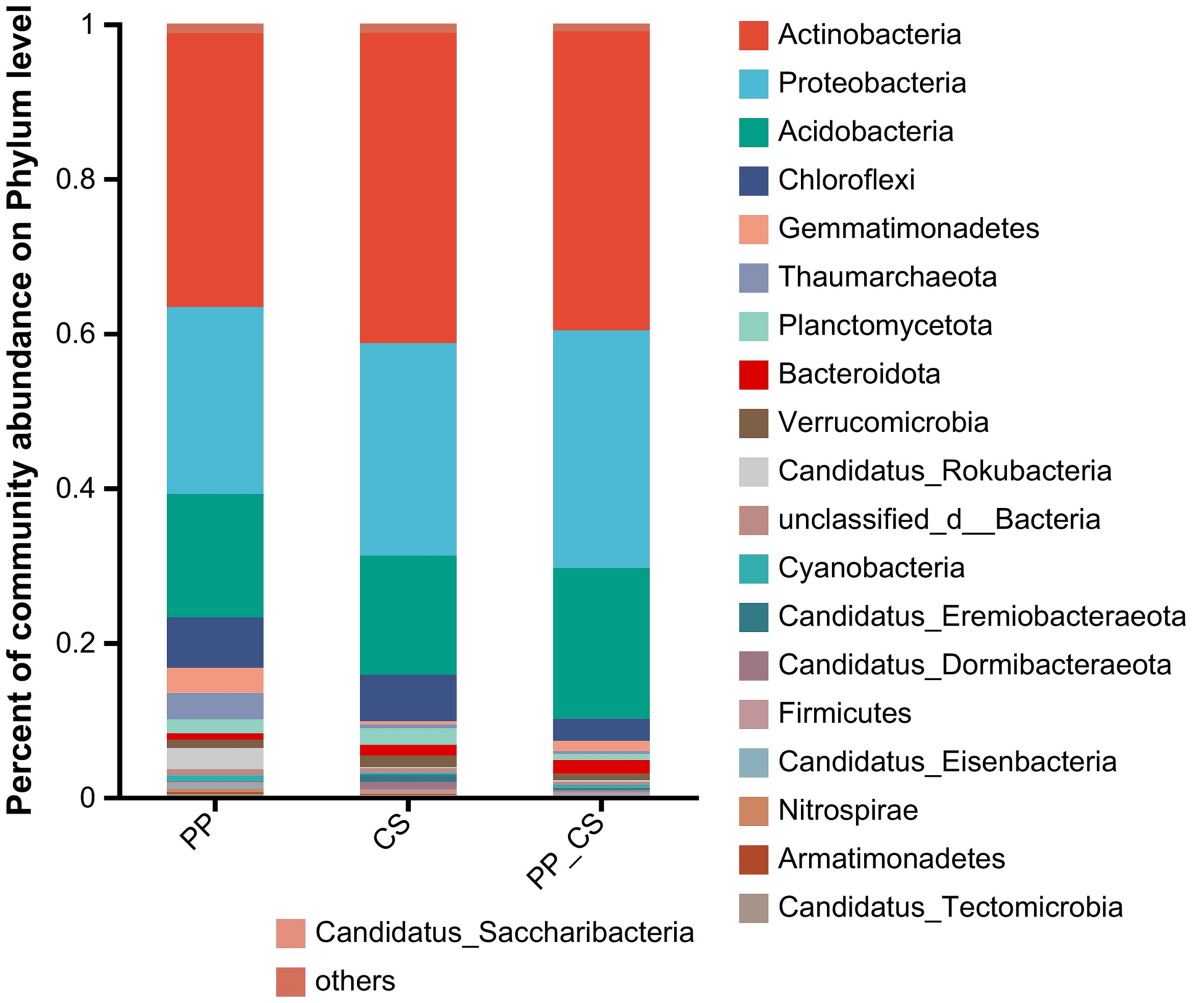
Stacked histogram of relative abundance of dominant phylum (top 20 in relative abundance) of bacterial communities.

Metagenomic sequencing revealed microbial taxa spanning one kingdom, 161 phyla, 269 classes, 485 orders, 1,000 families, 3,838 genera, and 27,624 species. Statistical analysis of the top 20 most abundant taxa demonstrated that Actinobacteria, Proteobacteria, Acidobacteria, and Chloroflexi dominated at the phylum level. Actinobacteria exhibited the highest relative abundance across all three experimental groups, emerging as the most dominant phylum. Although the dominant phyla remained consistent among experimental groups, their relative abundances differed significantly. For instance, Proteobacteria (30.7%) and Acidobacteria (19.48%) in PP_CS showed markedly higher abundances compared to PP (24.15%) and CS (27.47%), respectively (Figure 1).

**Figure 2:**
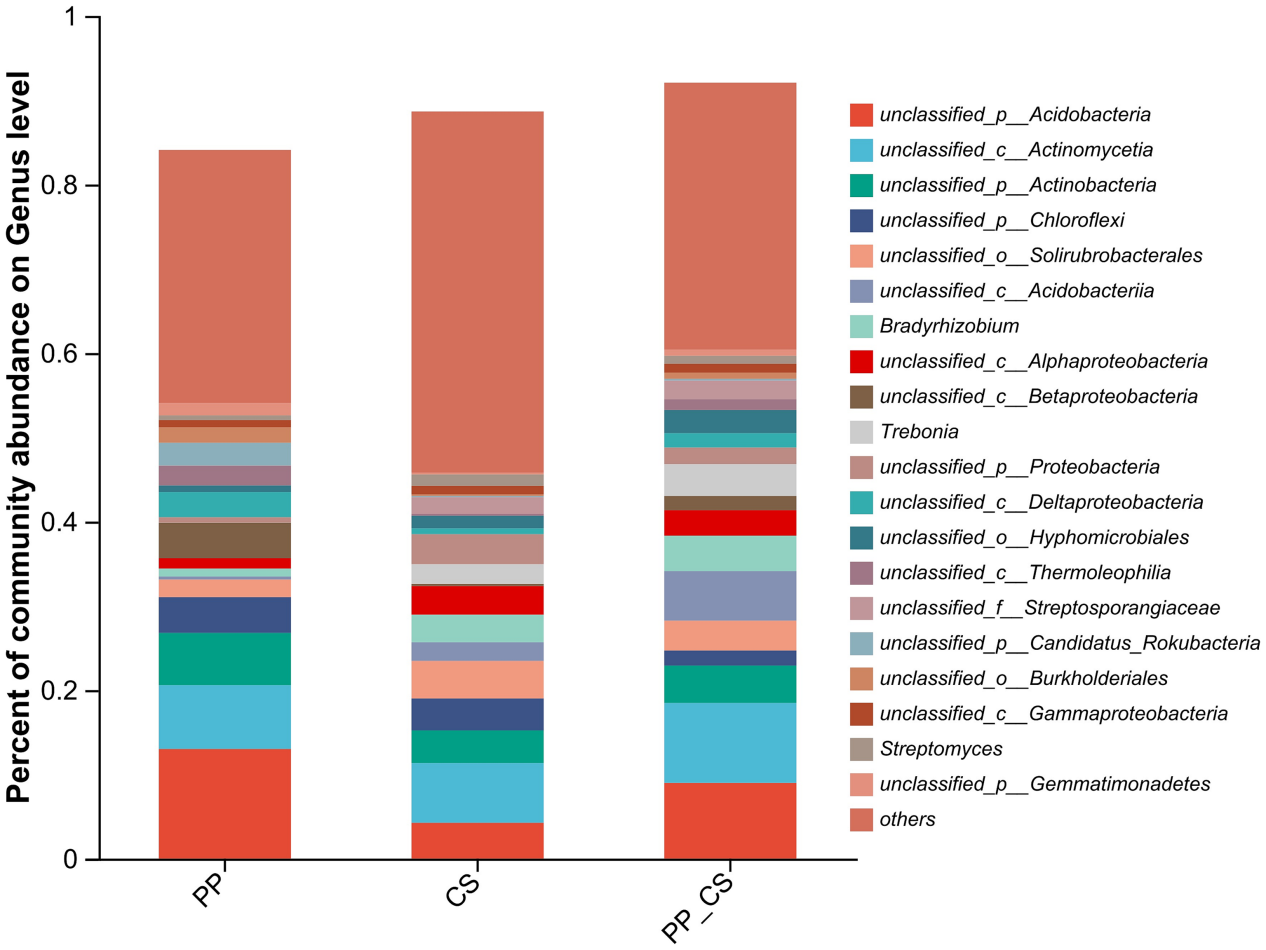
Soil microbial community relative percentage bar plot analysis at the genus level.

Distinct patterns of bacterial composition at the genus level were observed across the three experimental groups. In the PP group, predominant genera included unclassified_p__Acidobacteria, unclassified_c__Actinomycetia, unclassified_p__Actinobacteria, and unclassified_p__Chloroflexi. The CS group was dominated by unclassified_c__Actinomycetia (7.08%), unclassified_o__Solirubrobacterales (4.46%), unclassified_p__Acidobacteria (4.25%), and unclassified_p__Actinobacteria (3.87%). In contrast, the PP_CS group exhibited a distinct profile, with unclassified_p__Acidobacteria (8.98%), unclassified_c__Acidobacteriia (5.87%), unclassified_p__Actinobacteria (4.43%), *Bradyrhizobium* (4.20%), and *Trebonia* (3.78%) as dominant taxa (Figure 2).

**Figure 3:**
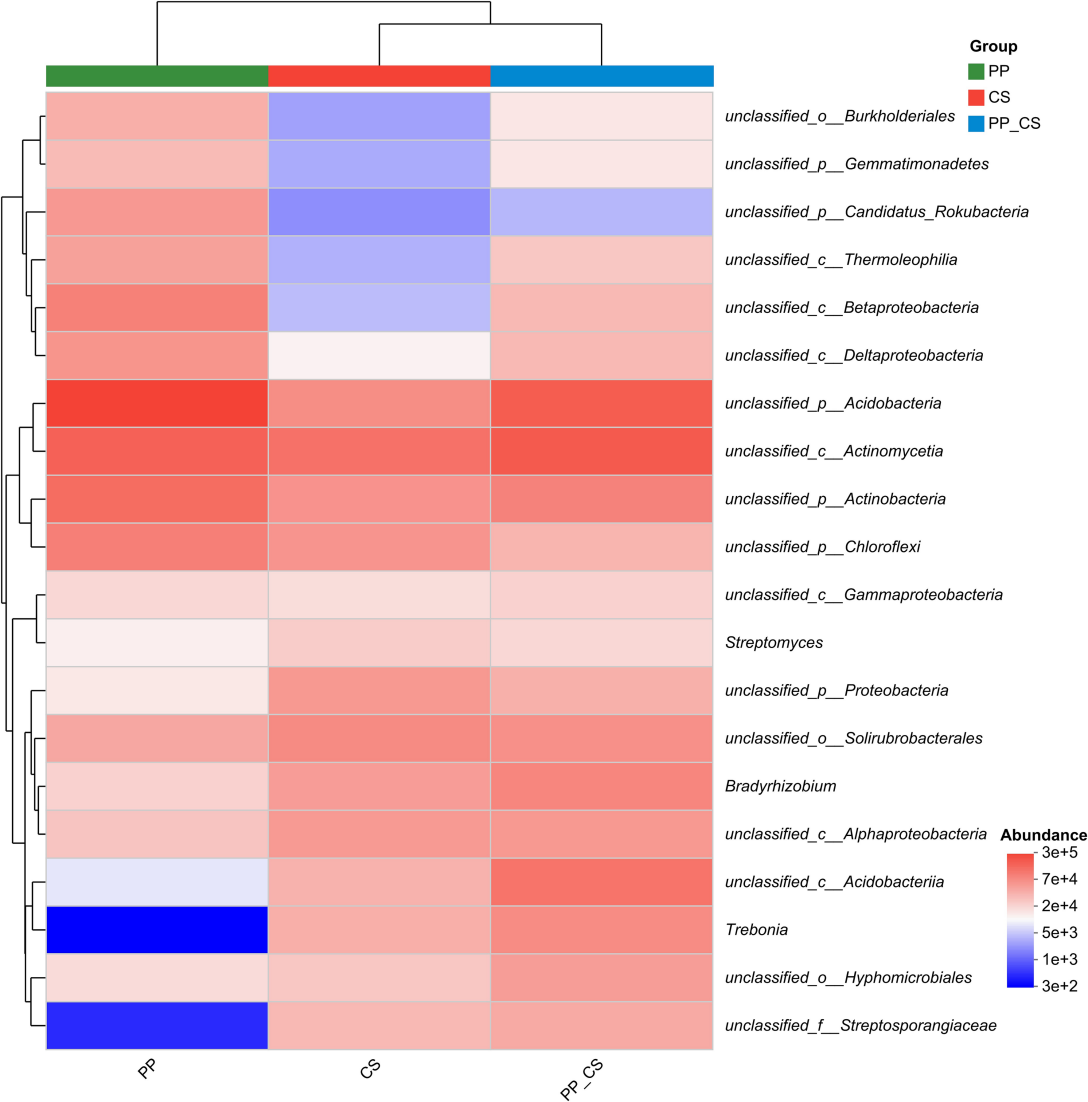
Community heatmap analysis of similarity in abundance between samples at the genus level (top 20 in relative abundance).

Cluster analysis of the top 20 microbial genera by absolute abundance revealed distinct grouping patterns at the genus level. The CS and PP_CS groups formed a closely related cluster, indicating higher microbial community similarity between these two groups compared to PP. As evidenced by color gradients in the heatmap reflecting taxon-specific abundance variations, distinct distribution patterns were observed across microbial communities. The CS and PP_CS groups exhibited heightened color intensity for *Streptomyces*, *Bradyrhizobium*, and *Trebonia*, corresponding to significantly higher relative abundances compared to the PP group (Figure 3).

### 3.2 Differential analysis of community composition

**Figure 4:**
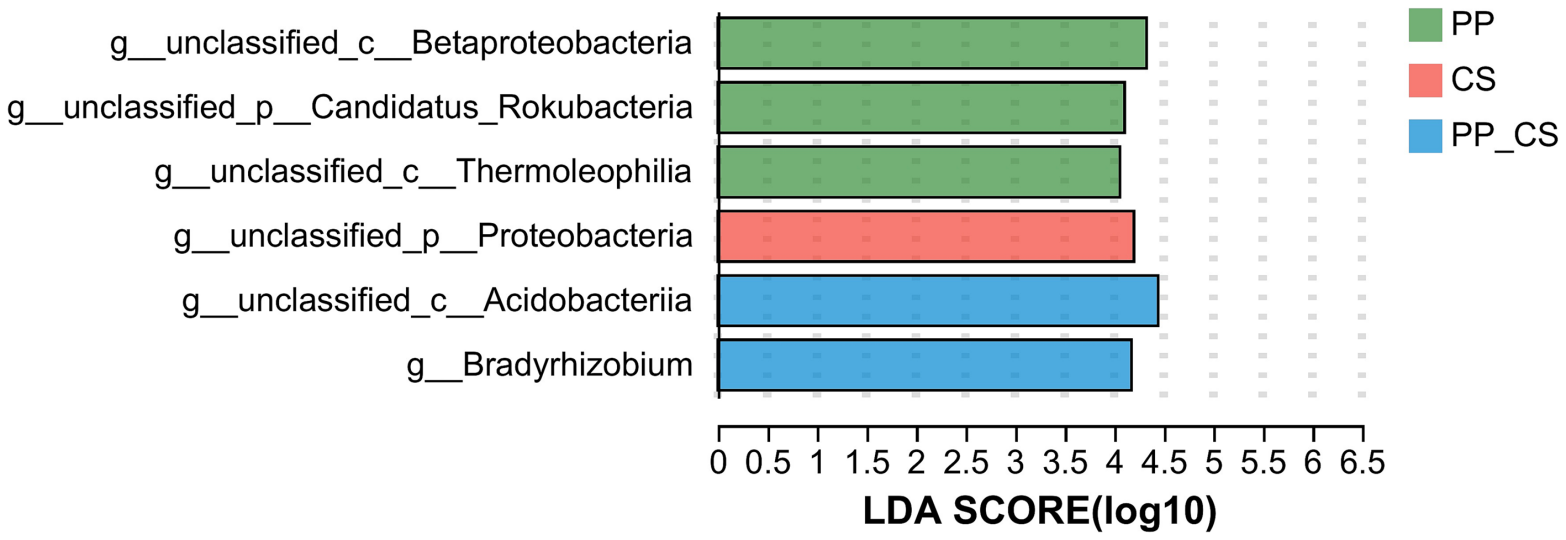
LDA discriminant histogram of flora at the genus level.

The genus exhibiting statistically significant differences among the three groups was identified using LEfSe (Linear Discriminant Analysis Effect Size), with genera possessing LDA scores greater than four selected for bar plot visualization. The analysis revealed three, one, and two significantly discriminant genera in the PP, CS, and PP_CS groups, respectively. Notably, unclassified_c__Acidobacteriia in the PP_CS group demonstrated the highest LDA score, followed by unclassified_c__Betaproteobacteria in the PP group (Figure 4).

### 3.3 Functional profiling of microbial communities

**Figure 5:**
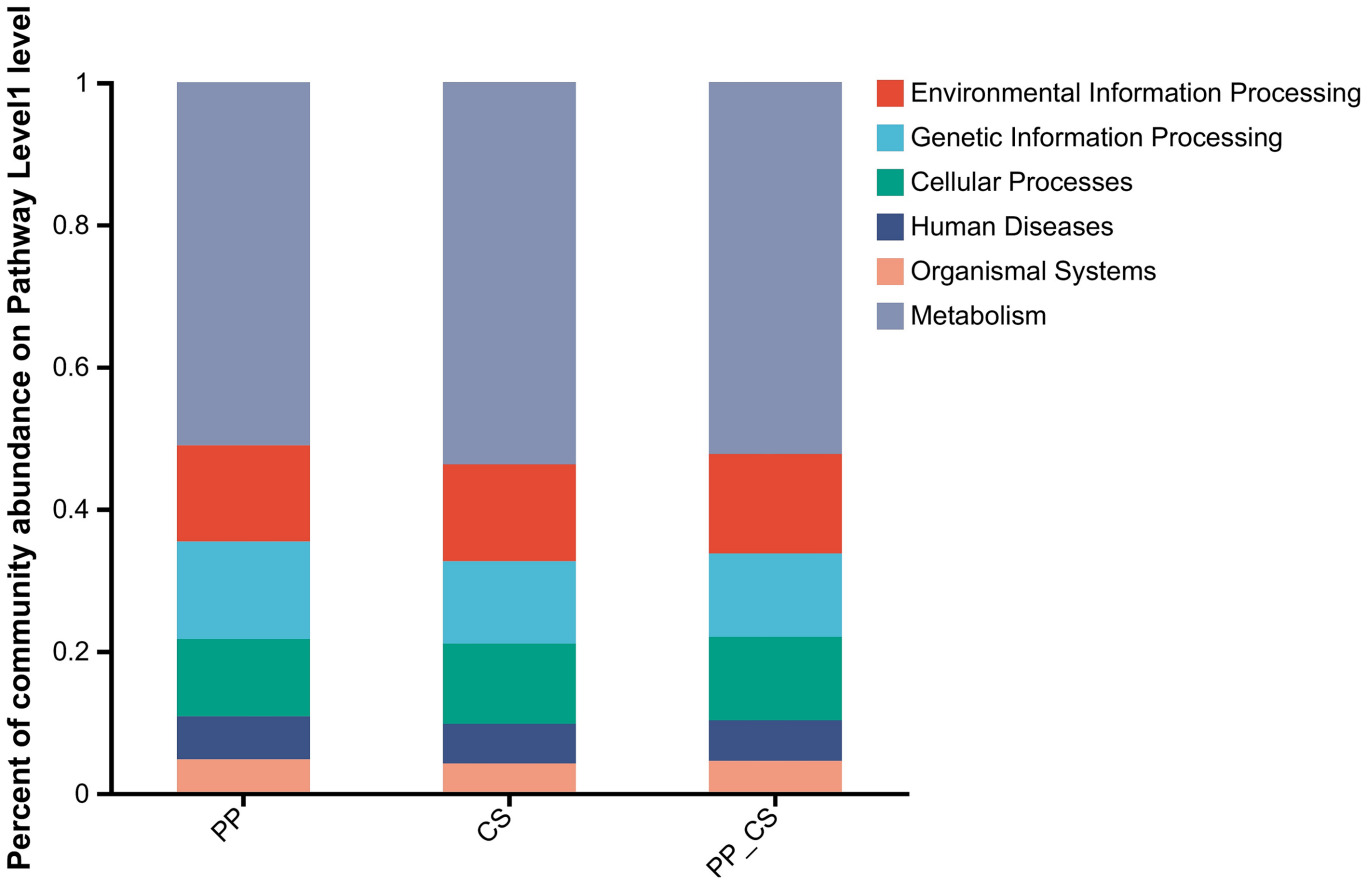
Histogram of relative abundance of functional composition of soil microbial communities under the KEGG level 1 database.

Functional annotation of the non-redundant gene set across soil samples was performed using the KEGG database. A total of 38,946,696 reads were successfully annotated, with 36,111,608 reads assigned to *metabolism*, 3,716,276 to *genetic information processing*, 4,312,784 to *environmental information processing*, 3,918,566 to *cellular processes*, 2,542,030 to *human diseases*, and 1,532,366 to *organismal systems*. At KEGG Level 1, *metabolism* demonstrated the highest relative abundance across all treatments, accounting for 51.07% (PP), 53.76% (CS), and 52.31% (PP_CS) of annotated functions, confirming its dominance in soil microbial activity. The *environmental information processing* and *cellular processes* reached peak abundance in the PP_CS group. *Genetic information processing*, *human diseases*, and *organismal systems* were most prevalent in PP. *Metabolism* exhibited maximal representation in CS (Figure 5).

**Figure 6:**
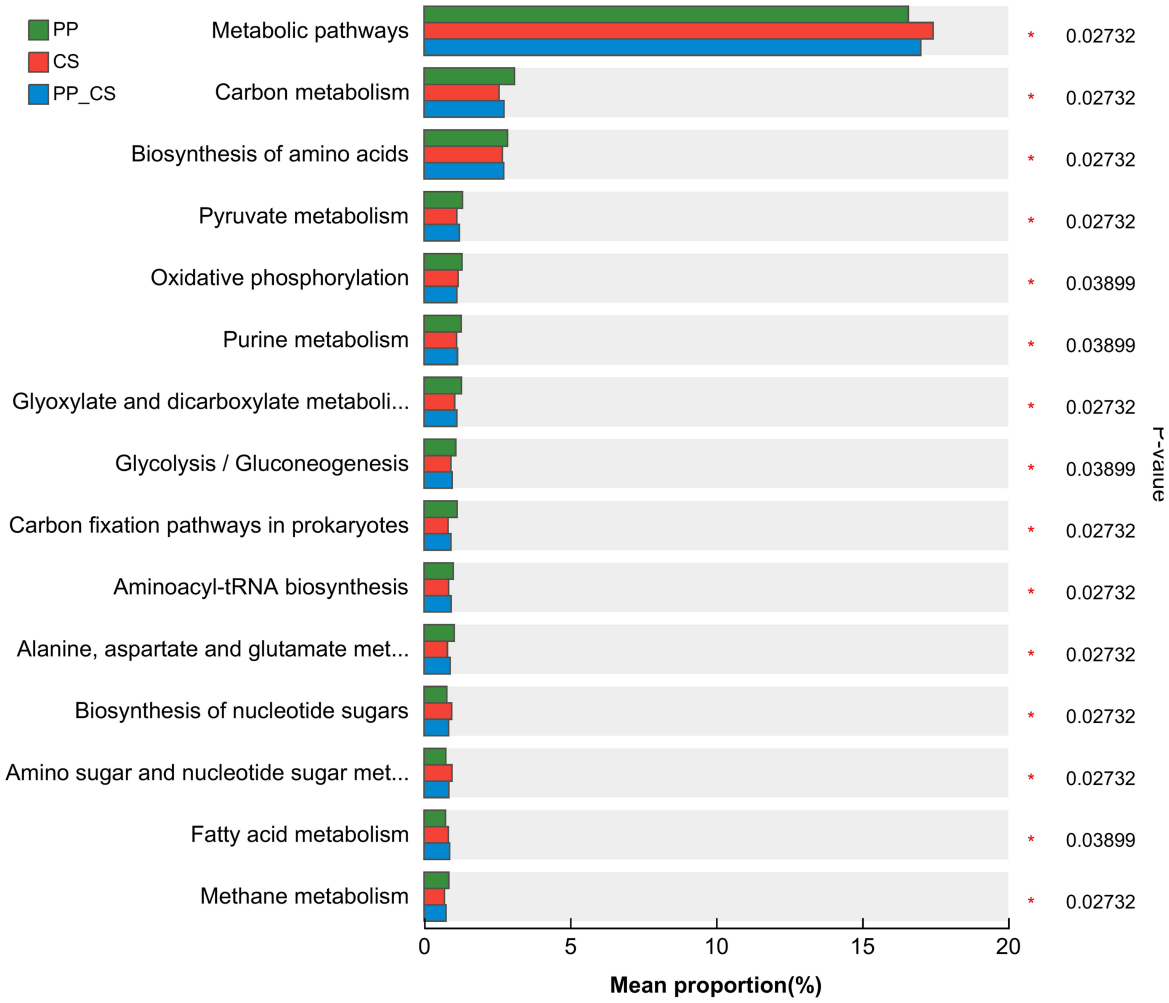
STAMP analysis of the relative abundance of functional genes at KEGG level 3 obtained under different soils.

The comparative analysis at KEGG level 3 revealed that *Metabolic pathways* constituted the most abundantly annotated functional category across all three groups, followed by *Carbon metabolism* and *Biosynthesis of amino acids*. Significant differences in microbial functional metabolism were observed among the different soil samples. Notably, CS groups exhibited higher relative abundances of *Metabolic pathways*, *Biosynthesis of nucleotide sugars*, and *Amino sugar and nucleotide sugar metabolism*. In contrast, PP_CS groups demonstrated enriched *Fatty acid metabolism*, while other predominant functional categories showed elevated abundances in PP groups (Figure 6).

### 3.4 Correlation analysis between soil functional composition and physicochemical factors

**Figure 7:**
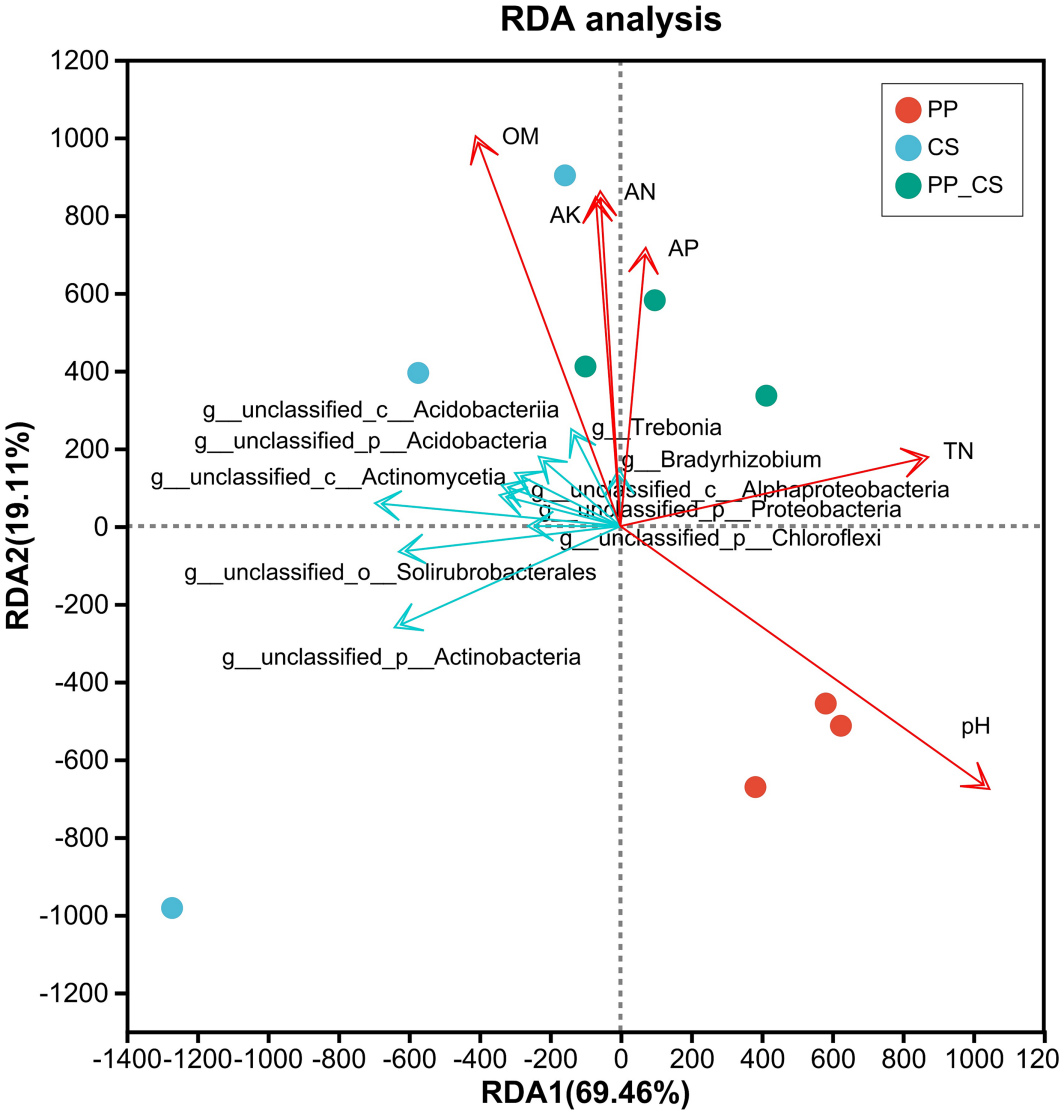
RDA between soil microbial functional composition and soil physicochemical properties in three soils.

The RDA is a type of PCA analysis that is constrained by environmental factors. For the pictorial representation of the relationship between microbial flora at the genus level and environmental factors, samples and environmental factors were drawn on the same 2-D sequence diagram (Huhe et al., 2017). The results demonstrated significant impacts of cultivation regimes on soil microbial functional composition. The horizontal and vertical ordination axes explained 67.77 and 19.11% of the functional variation, respectively, indicating the representativeness of the selected physicochemical parameters of soils. The proximity of CS and PP_CS samples, in contrast to their marked separation from PP samples, reflects distinct microbial functional profiles between these groups. Among the five analyzed soil properties, organic matter (OM) showed positive correlations with alkali hydrolyzed nitrogen (AN), available phosphorus (AP), and available potassium (AK), while total nitrogen (TN) showed a positive correlation with pH. Furthermore, correlations among soil bioactive components, microbial taxa, and cultivation systems were investigated. Notably, pH exhibited stronger associations with monoculture loquat (PP) soils, OM content was closely linked to monoculture tea (CS) soils, whereas AP and AK exerted more pronounced influences on tea-fruit intercropped (PP_CS) soils (Figure 7).

Within the PP_CS group, *Trebonia* and *Bradyrhizobium* demonstrated significant correlations with OM, AP, and AK, while pH and TN showed no associations with these dominant genera. These findings collectively suggest that edaphic properties have a significant influence on the composition of intercropped soil microbial communities.

## 4 Discussion

Soil microbial community composition is predominantly shaped by soil type and plant species, with key physicochemical parameters, including organic matter content and nitrogen-phosphorus-potassium (NPK) concentrations, critically influencing rhizosphere microbial abundance, community structure, and functional dynamics, particularly in modulating microbial functional diversity. Reciprocally, soil microorganisms drive critical processes such as organic matter decomposition, humification, nutrient transformation, and biogeochemical cycling, serving as the primary catalytic force for soil organic matter turnover and nutrient fluxes (Eduardo et al., 2015). This bidirectional interaction between soil nutrient environments and rhizosphere microbiota collectively sustains the stability of plant-soil systems. Diversified intercropping systems not only maintain microbial equilibrium in soil ecosystems but also significantly enhance microbial biomass, metabolic activity, and biodiversity (Ma et al., 2017). Through metagenomic analysis of rhizosphere soils under three cultivation regimes, this study revealed high community and functional diversity. At the phylum level, Actinobacteria, Proteobacteria, Acidobacteria, and Chloroflexi emerged as dominant taxa. Notably, loquat-tea intercropping significantly increased the abundance of Bacteroidota in tea rhizospheres compared to monocultures. Given that Bacteroidota are recognized as pivotal contributors to soil nutrient transformation, their enrichment in intercropped systems likely enhances nutrient acquisition efficiency (Yousuf et al., 2012).

Furthermore, field applications of plant growth-promoting rhizobacteria have been shown to elevate Bacteroidota abundance, suggesting a viable strategy for optimizing soil nutrient management (Hou et al., 2015). Intriguingly, both intercropped and monoculture systems in this study exhibited Ascomycota as the predominant fungal phylum, followed by Mortierellomycota, a pattern consistent with observations in the rhizospheres of *Pinellia ternata* and maize (Huang et al., 2022). These findings underscore the sensitivity of soil microbial communities in disease-prone soils, where shifts in diversity and structure can have a direct impact on soil-borne pathogen dynamics (Jiao et al., 2022).

At the genus level, monoculture soils were dominated by unclassified Acidobacteria, unclassified Actinomycetia, and unclassified Actinobacteria. In contrast, intercropping enriched functional genera, including *Streptomyces*, *Bradyrhizobium*, *Trebonia*, and *Agromyces*. *Streptomyces* spp., phosphate-solubilizing actinobacteria with biocontrol properties, enhance phosphorus availability via enzymatic mineralization of organic phosphates (Gao et al., 2020; Li and Shi, 2006). *Agromyces* serves as a bioindicator of soil health, promoting plant growth through multifaceted interactions (Wongkiew et al., 2022; Hu et al., 2021). *Sphingomonas*, characterized by glycosphingolipid-enriched membranes, exhibits exceptional resilience under nutrient-limited conditions and demonstrates bioremediation potential through the degradation of recalcitrant organic pollutants (Li et al., 2021). Intercropping also increased the relative abundance of Arthrographis, a fungal genus associated with humus formation. Compost-derived humus not only improves soil structure but also suppresses phytopathogens, highlighting the dual benefits of intercropping in enhancing both microbial functionality and plant health (Zhang et al., 2022). It can be seen that intercropping loquat significantly increased the microbial abundance, which is beneficial to the healthy growth of tea plants.

The composition-structure-function continuum of soil microbial communities is highly responsive to environmental perturbations, where shifts in species diversity may cascade into functional alterations (He et al., 2022). Level 1 KEGG pathway analysis revealed predominant microbial engagement in *metabolism*, *genetic information processing*, *human diseases*, and *cellular processes*, corroborating the intense metabolic activity within soil ecosystems. Chen Q. L. et al. (2020) and Chen Y. et al. (2020) identified microbial functional gene abundance as the primary determinant of soil process rates (e.g., respiration, nitrification/denitrification), while Xiang et al. (2020) established significant positive correlations between carbon-nitrogen-phosphorus-sulfur functional gene abundance and microbial diversity, suggesting that functional gene profiles reflect community structural complexity. Soil microbial metabolism is conducive to maintaining the stability and functionality of the soil ecosystem. It can not only improve soil structure by increasing soil porosity, but also provide energy sources for microorganisms and maintain the growth and reproduction of soil microorganisms (Wang et al., 2024). Intercropped soils demonstrated the highest *functional metabolic* abundance, indicating that intercropping regimes enhance soil microbial metabolic capacity, which in turn facilitates plant nutrient acquisition. This *metabolism* function enrichment may stem from increased plant diversity-driven humification processes, where heightened humus production necessitates extensive compound transformation. Microbial decomposition of these compounds requires energy-intensive metabolic activities, thereby promoting the proliferation of metabolically active communities—a mechanism reflecting microbial adaptive strategies to environmental stressors (Toledo et al., 2017). The function of the soil ecosystem mainly depends on the functional diversity, which is more mediated by the rhizosphere-specific functional flora (Lakshmanan et al., 2014). A large number of studies have shown that soil actinobacteria and Firmicutes bacteria have the functions of inhibiting the growth of pathogens, activating plant immunity, promoting plant growth, degrading complex organic matter, and enhancing the soil nutrient cycle (Lee et al., 2021; Chen Q. L. et al., 2020; Chen Y. et al., 2020). *Streptomyces*, *Bacillus*, *Bradyrhizobium*, *Trebonia*, and *Agromyces* are commonly reported plant growth-promoting bacteria or biocontrol bacteria, which play an important role in maintaining soil health (Li T. et al., 2018; Li M. S. et al., 2018). Studies have also shown that *Streptomyces*, *Bacillus*, and *Arthrobacter* also have biological nitrogen fixation, phosphate solubilization, and potassium solubilization functions (Ali et al., 2021). The results of microbial species obtained in the above study are basically consistent with the results of this study, indicating that the microbial activities after tea-loquat intercropping can effectively promote the rapid cycling of soil nutrients, improve the activation of nutrients, and inhibit pathogens. In addition, microbial function analysis also found that the relative abundance of functional categories such as *fatty acid metabolism* and c*arbon metabolism* of rhizosphere microbial community in PP_CS group was higher than that in PP and CS group, which may be related to the increase of the type and content of organic matter such as root exudates after tea-loquat intercropping, which improved the metabolic activity and function of rhizosphere microorganisms. Soil microbial carbon metabolism has an important impact on the function and stability of the soil ecosystem. It can not only improve soil structure and increase soil porosity, but also provide energy sources for soil microorganisms to maintain microbial growth and reproduction. Therefore, the increase in the abundance of beneficial bacteria after tea fruit intercropping may help to reduce the negative feedback effect of plant soil, accelerate nutrient circulation and supply, maintain soil health, and promote the growth of intercropping plants.

Soil organic matter is a kind of carbon containing organic compounds in various forms and states in soil, in which humus is the main body of organic matter. Nitrogen, phosphorus, and potassium are essential nutrients in the ecosystem (Agnan et al., 2019). Extensive studies have documented the fertility-enhancing effects of intercropping, particularly in elevating organic matter, nitrogen, and phosphorus levels compared to monocultures (Brooker et al., 2015; Wu et al., 2020; Xiao et al., 2013). Zhu et al. (2006) reported superior tea quality and soil nutrient status in persimmon-tea intercropping systems, while Li et al. (2022) demonstrated significant increases in total phosphorus and potassium in chestnut-tea intercropped rhizospheres. Remarkably, three-year cassava-soybean intercropping elevated available NPK concentrations by ~20-fold (Tang et al., 2020). This study found that the correlation between the PP_CS group and OM, AN, AK, and AP was high. The results were basically consistent with previous studies, indicating that tea-loquat intercropping, like other crop intercropping, improved soil organic matter and nutrients, accelerated the transformation of nutrient elements, promoted the utilization of nitrogen, phosphorus, and potassium by plants, and was beneficial to the health of rhizosphere soil. It was found that the structure of the soil microbial population changed and the abundance increased with the application of nitrogen, phosphorus, and potassium fertilizers (Enebe and Babalola, 2020). Our study further revealed strong positive correlations between intercropping and rhizosphere AN, AP, and AK levels, with *Trebonia* (a novel acidophilic actinobacterium with biocontrol efficacy against soil-borne diseases) (Rapoport et al., 2020) and *Bradyrhizobium* (a well-documented plant growth promoter in legumes) showing tight linkages to OM, AN, AP, and AK dynamics (Agha et al., 2023). These findings advocate for the strategic management of organic fertilizers, AP, and AK in loquat-tea intercropping systems to synergistically enhance soil fertility and beneficial microbiota.

## 5 Conclusion

Our analysis of rhizosphere soil microbial communities in tea-fruit intercropping systems revealed three dominant phyla across all soil types: Actinobacteria, Proteobacteria, and Acidobacteria. Notably, loquat intercropping significantly enhanced the abundance of Bacteroidota in tea rhizospheres compared to monoculture systems. At the genus level, intercropping markedly increased the relative abundance of beneficial taxa, including *Streptomyces*, *Bradyrhizobium*, *Trebonia*, and *Agromyces*, establishing a rhizospheric environment conducive to suppressing root-associated phytopathogens. Distinct functional compositional shifts were observed between intercropped and monoculture soils, with intercropped systems exhibiting the highest functional abundance in metabolism pathways, indicative of enhanced microbial metabolic capacity. The RDA identified organic matter (OM), alkali hydrolyzed nitrogen (AN), available phosphorus (AP), and available potassium (AK) as critical drivers of microbial functional composition, with AP and AK exerting the most pronounced effects.

## Data Availability

Publicly available datasets were analyzed in this study. This data can be found here: the datasets presented in this study are available in the online NCBI repository with accession number(s) SRP590738.

## Footnotes

^1^ https://github.com/OpenGene/fastp

^2^ https://github.com/voutcn/megahit

^3^ http://metagene.cb.k.u-tokyo.ac.jp/

^4^ http://www.bioinformatics.org/cd-hit

^5^ http://soap.genomics.org.cn/

^6^ http://blast.ncbi.nlm.nih.gov/Blast.cgi

^7^ https://ftp.ncbi.nlm.nih.gov/blast/db/FASTA/

^8^ https://github.com/bbuchfink/diamond

## References

[ref1] Abdul Rauf C. Naz F. Ahmad I. Ul Haque I. Riaz A. (2015). Management of black scurf of potato with effective microbes (EM), biological potassium fertilizer (BPF) and Trichoderma harzianum. Int. J. Agric. Biol. 17, 601–606. doi: 10.17957/IJAB/17.3.14.019

[ref2] Agha M. S. Haroun S. Abbas M. A. Haroun S. A. Sofy M. R. Mowafy A. M. (2023). Growth and metabolic response of *Glycine max* to the plant growth-promoting *Enterobacter* Delta PSK and *Bradyrhizobium japonicum* under salinity stress. J. Plant Growth Regul. 42, 5816–5830. doi: 10.1007/s00344-023-10967-4

[ref3] Agnan Y. Courault R. Alexis M. A. Zanardo T. Cohen M. Sauvage M.. (2019). Distribution of trace and major elements in subarctic ecosystem soils: sources and influence of vegetation. Sci. Total Environ. 682, 650–662. doi: 10.1016/j.scitotenv.2019.05.178 31129547

[ref4] Ali A. M. Awad M. Y. M. Hegab S. A. El Gawad A. M. A. Eissa M. A. (2021). Effect of potassium solubilizing bacteria (*Bacillus cereus*) on growth and yield of potato. J. Plant Nutr. 44, 411–420. doi: 10.1080/01904167.2020.1822399

[ref5] Brooker R. W. Bennett A. E. Cong W. F. Daniell T. J. George T. S. Hallett P. D.. (2015). Improving intercropping: a synthesis of research in agronomy, plant physiology and ecology. New Phytol. 206, 107–117. doi: 10.1111/nph.13132, PMID: 25866856

[ref6] Burke C. Steinberg P. Rusch D. Kjelleberg S. Thomas T. (2011). Bacterial community assembly based on functional genes rather than species. Proc. Natl. Acad. Sci. U.S.A. 108, 14288–14293. doi: 10.1073/pnas.1101591108, PMID: 21825123 PMC3161577

[ref7] Chen Y. Bonkowski M. Shen Y. Griffiths B. S. Jiang Y. J. Wang X. Y.. (2020). Root ethylene mediates rhizosphere microbial community reconstruction when chemically detecting cyanide produced by neighbouring plants. Microbiome 8:4. doi: 10.1186/s40168-019-0775-6, PMID: 31954405 PMC6969408

[ref8] Chen Q. L. Ding J. Li C. Y. Yan Z. Z. He J. Z. Hu H. W. (2020). Microbial functional attributes, rather than taxonomic attributes, drive top soil respiration, nitrification and denitrification processes. Sci. Total Environ. 734:139479. doi: 10.1016/j.scitotenv.2020.139479, PMID: 32464393

[ref9] Chen H. S. Qin C. X. Peng C. Guo Q. Tang L. Q. Chen Y. Q.. (2019). Effects of sugarcane and peanut intercropping on rhizosphere soil microbial populations and enzyme activities. Jiangsu Agric. Sci. 47, 223–226. doi: 10.15889/j.issn.1002-1302.2019.03.053

[ref10] Chen C. H. Wang Y. Tang Q. Chen S. X. (2011). Analysis of ecological and economic effects of tea garden intercropping with pear trees. Southwest China J. Agric. Sci. 24, 1446–1449. doi: 10.3969/j.issn.1001-4829.2011.04.045

[ref11] Duchene O. Vian J. F. Celette F. (2017). Intercropping with legume for agroecological cropping systems: complementarity and facilitation processes and the importance of soil microorganisms: a review. Agric. Ecosyst. Environ. 240, 148–161. doi: 10.1016/j.agee.2017.02.019

[ref12] Eduardo P. V. Marta G. Miguel V. (2015). Phylogenetic structure of soil bacterial communities predicts ecosystem functioning. FEMS Microbiol. Ecol. 91:fiv031. doi: 10.1093/femsec/fiv031 25873469

[ref13] Enebe M. C. Babalola O. O. (2020). Effects of inorganic and organic treatments on the microbial community of maize rhizosphere by a shotgun metagenomics approach. Ann. Microbiol. 70:49. doi: 10.1186/s13213-020-01591-8

[ref14] Fierer N. Leff J. W. Adams B. J. Nielsen U. N. Bates S. T. Lauber C. L.. (2012). Cross-biome metagenomic analyses of soil microbial communities and their functional attributes. Proc. Natl. Acad. Sci. U.S.A. 109, 21390–21395. doi: 10.1073/pnas.1215210110, PMID: 23236140 PMC3535587

[ref15] Fu W. Wu H. Zhao A.-H. Hao Z.-P. Chen B.-D. (2020). Ecological impacts of nitrogen deposition on terrestrial ecosystems: research progresses and prospects. Chin. J. Plant Ecol. 44, 475–493. doi: 10.17521/cjpe.2019.0163

[ref16] Gao G. Bo D. Ke C. Qin H. (2020). Identification of a *Streptomyces* phosphorus-solubilizing strain and the factors affecting phosphorus-solubilizing ability. J. Northeast For. Univ. 48, 102–109. doi: 10.13759/j.cnki.dlxb.2020.01.018

[ref17] He Z. S. Chen J. J. Zhu J. Wang Z. W. Gu X. G. Jiang L.. (2022). Characteristics of microbial functional diversity and its influencing factors of forest soils at different elevations on the southern slope of Daiyun Mountain. Acta Ecol. Sin. 42, 3504–3515. doi: 10.5846/stxb202101210217

[ref18] Hou J. Y. Liu W. X. Wang B. B. Hou J. Liu W. Wang B.. (2015). PGPR enhanced phytoremediation of petroleum contaminated soil and rhizosphere microbial community response. Chemosphere 138, 592–598. doi: 10.1016/j.chemosphere.2015.07.025, PMID: 26210024

[ref19] Hu H. Y. Li H. Hao M. M. Ren Y. N. Zhang M. K. Liu R. Y.. (2021). Nitrogen fixation and crop productivity enhancements co-driven by intercrop root exudates and key rhizosphere bacteria. J. Appl. Ecol. 58, 2243–2255. doi: 10.1111/1365-2664.13964

[ref20] Huang Z. Y. Zhao G. R. Liu T. Wu J. X. Xu R. Cun M. Z.. (2022). Effects of intercropping *Pinellia ternata* with maize on rhizosphere microbial community. Mol. Plant Breed., 1–13.

[ref9001] Huhe C. X. Hou F. Wu Y. Cheng Y. (2017). Bacterial and fungal community structures in loess plateau grasslands with different grazing intensities. Front. Microbiol. 8:606. doi: 10.3389/fmicb.2017.00606 28439265 PMC5383705

[ref21] Jiao N. Song X. Song R. Yin D. Deng X. (2022). Diversity and structure of the microbial community in rhizosphere soil of *Fritillaria ussuriensis* at different health levels. PeerJ 10:e12778. doi: 10.7717/peerj.12778, PMID: 35127284 PMC8796711

[ref22] Lakshmanan V. Selvaraj G. Bais H. P. (2014). Functional soil microbiome: belowground solutions to an aboveground problem. Plant Physiol. 166, 689–700. doi: 10.1104/pp.114.245811, PMID: 25059708 PMC4213098

[ref23] Lange M. Habekost M. Eisenhauer N. Roscher C. Bessler H. Engels C.. (2014). Biotic and abiotic properties mediating plant diversity effects on soil microbial communities in an experimental grassland. PLoS One 9:e96182. doi: 10.1371/journal.pone.0096182, PMID: 24816860 PMC4015938

[ref24] Lee S. M. Kong H. C. Song G. C. Ryu C. M. (2021). Disruption of Fimicutes and Actinobacteria abundance in tomato rhizosphere causes the incidence of bacterial wilt disease. ISME J. 15, 330–347. doi: 10.1038/S41396-020-00785-X 33028974 PMC7852523

[ref25] Li L. (2016). Intercropping enhances agroecosystem services and functioning: current knowledge and perspectives. Chin. J. Eco-Agric. 24, 403–415. doi: 10.13930/j.cnki.cjea.160061

[ref26] Li M. S. Guo R. Yu F. Chen X. Zhao H. Y. Li H. X.. (2018). Indole-3-acetic acid biosynthesis pathways in the plant-beneficial bacterium *Arthrobacter pascens* ZZ21. Int. J. Mol. Sci. 19:443. doi: 10.3390/ijms19020443, PMID: 29389906 PMC5855665

[ref27] Li Q. Li J. Jiang L. Sun Y. Luo C. Zhang G. (2021). Diversity and structure of phenanthrene degrading bacterial communities associated with fungal bioremediation in petroleum contaminated soil. J. Hazard. Mater. 403:123895. doi: 10.1016/j.jhazmat.2020.123895, PMID: 33264959

[ref28] Li M. Liu L. Dao M. Yang Z. Wu T. (2022). Analysis of soil chemical properties and bacterial richness in chestnut-tea intercropping tea orchard. Non-Wood For. Res. 40, 58–65. doi: 10.14067/j.cnki.1003-8981.2022.01.007

[ref29] Li W. H. Shi J. Y. (2006). Isolation purification and phosphate-solubilizing of phosphorous bacteria in West Lake sediment. Chin. J. Appl. Ecol. 11, 2112–2116. doi: 10.1360/yc-006-1280 17269337

[ref30] Li T. Zhang W. Liu G. X. Chen T. (2018). Advances in the study of microbial ecology in desert soil. J. Desert Res. 38, 329–338. doi: 10.7522/j.issn.1000-694X.2017.00113

[ref31] Liu X. D. Bi C. H. Tan J. P. Kong X. J. (2016). Effect of chestnut-tea intercropping and covering straw on the tea tree growth environment and quality of tea. J. Anhui Agric. Sci. 44, 26–27. doi: 10.13989/j.cnki.0517-6611.2016.34.010

[ref32] Liu T. Dong M. Zhang L. Gu J. Zhang G. Qian H. (2017). Effects of different intercropping patterns on tea-planted soil and tea nutritional quality. J. Food Sci. Technol. 35, 67–76. doi: 10.3969/j.issn.2095-6002.2017.06.011

[ref33] Lu Y. Chen J. Zheng J. Hu G. Wang J. Huang C.. (2016). Mucosal adherent bacterial dysbiosis in patients with colorectal adenomas. Sci. Rep. 6:26337. doi: 10.1038/srep26337, PMID: 27194068 PMC4872055

[ref34] Luo Z. M. Liu J. X. Hu Y. Q. He L. Zhou Y. Y. Zheng Q. R.. (2023). Taxonomic and functional diversity of soil microbial communities in subalpine meadow with different degradation degrees in mount Wutai. Environ. Sci. 44, 2918–2927. doi: 10.13227/j.hjkx.202204330, PMID: 37177963

[ref35] Ma Y. H. Fu S. L. Zhang X. P. Zhao K. Chen H. Y. H. (2017). Intercropping improves soil nutrient availability, soil enzyme activity and tea quantity and quality. Appl. Soil Ecol. 119, 171–178. doi: 10.1016/j.ap-soil.2017.06.028

[ref36] Ma K. Yang G. L. Ma L. Wang C. M. Wei C. H. Dai X. H.. (2016). Effects of intercropping on soil microbial communities after long-term potato monoculture. Acta Ecol. Sin. 36, 2987–2995. doi: 10.5846/stxb201412072425

[ref37] Ma H. X. Zhang L. L. Sun X. M. Zhang H. Q. He M. X. Chen G. J.. (2015). Understanding microbial communities and their functions by meta-omics approaches. Microbiol. China 42, 902–912. doi: 10.13344/j.microbiol.china.140965

[ref38] Qin X. M. Huang S. X. Wei J. J. Li J. T. Chen X. Luo Y. F.. (2019). Effects of tea and soybean intercropping on tea-planted soil and tea nutritional quality. Acta Agric. Boreali-Sin. 34, 129–135. doi: 10.7668/hbnxb.20190261

[ref39] Qin X. M. Zheng Y. Tang L. Long G. (2017). Crop rhizospheric microbial community structure and functional diversity as affected by maize and potato intercropping. J. Plant Nutr. 40, 2402–2412. doi: 10.1080/01904167.2017.1346674

[ref40] Rapoport D. Sagova-Mareckova M. Sedlacek I. Provaznik J. Kralova S. Pavlinic D.. (2020). *Trebonia kvetii* gen. nov., sp. nov., an acidophilic actinobacterium, and proposal of the new actinobacterial family *Treboniaceae* fam. nov. Int. J. Syst. Evol. Microbiol. 70, 5106–5114. doi: 10.1099/ijsem.0.004388 32804604

[ref41] Tang X. M. Zhong R. C. Jiang J. Tang X. Zhong R. He L.. (2020). Cassava/peanut intercropping improves soil quality via rhizospheric microbes increased available nitrogen contents. BMC Biotechnol. 20:13. doi: 10.1186/s12896-020-00606-1, PMID: 32111197 PMC7049180

[ref42] Toledo M. Gutiérrez M. C. Siles J. A. García-Olmo J. Martín M. A. (2017). Chemometric analysis and NIR spectroscopy to evaluate odorous impact during the composting of different raw materials. J. Clean. Prod. 167, 154–162. doi: 10.1016/j.jclepro.2017.08.163, PMID: 40698065

[ref43] van der Heijden M. G. A. Bardgett R. D. van Straalen N. M. (2008). The unseen majority: soil microbes as drivers of plant diversity and productivity in terrestrial ecosystems. Ecol. Lett. 11, 296–310. doi: 10.1111/j.1461-0248.2007.01139.x, PMID: 18047587

[ref45] Wang S. Q. Li R. P. Song L. Z. Pan C. L. Yang Z. Y. Liang D. J. (2024). Effects of intercropping *Belamcanda chinensis* on the structure and function of soil bacterial community in poplar forest land. North. Hortic. 19, 81–89. doi: 10.11937/bfyy.20240779

[ref46] Wang L. Liu J. Chai B. (2022). Response of soil bacterial community and nitrogen cycle during the natural recovery of abandoned farmland in subalpine of the North China. Ecol. Environ. Sci. 31, 1537–1546. doi: 10.16258/j.cnki.1674-5906.2022.08.005

[ref47] Wang Z. J. Wang S. Liu Y. Y. Feng K. Deng Y. (2018). The applications of metagenomics in the detection of environmental microbes involving in nitrogen cycle. Biotechnol. Bull. 34, 1–14. doi: 10.13560/j.cnki.biotech.bull.1985.2018-0024

[ref48] Wang R. Xiao M. Li D. Ling T. Xie Z. (2018). Recent advance on quality characteristics and health effects of dark tea. J. Tea Sci. 38, 113–124. doi: 10.13305/j.cnki.jts.2018.02.001

[ref49] Wongkiew S. Chaikaew P. Takrattanasaran N. Khamkajorn T. (2022). Evaluation of nutrient characteristics and bacterial community in agricultural soil groups for sustainable land management. Sci. Rep. 12:7368. doi: 10.1038/s41598-022-09818-1, PMID: 35513414 PMC9072534

[ref50] Wu B. Feng K. Zhi X. Y. He Q. Xu M. Y. Deng Y.. (2017). Progresses in environmental microbiome diversity and function research. Acta Sci. Nat. Univ. Sunyatseni 56, 1–11. doi: 10.13471/j.cnki.acta.snus.2017.05.001

[ref51] Wu X. Zhang T. Zhao J. N. Zhao J. Wang L. Yang D.. (2020). Variation of soil bacterial and fungal communities from fluvo-aquic soil under chemical fertilizer reduction combined with organic materials in North China plain. J. Soil Sci. Plant Nutr. 21, 349–363. doi: 10.1007/s42729-020-00365-0

[ref52] Xiang Q. Chen Q. L. Zhu D. Yang X. R. Qiao M. Hu H. W.. (2020). Microbial functional traits in phyllosphere are more sensitive to anthropogenic disturbance than in soil. Environ. Pollut. 265:114954. doi: 10.1016/j.envpol.2020.114954, PMID: 32544665

[ref53] Xiao X. M. Cheng Z. H. Meng H. W. Xiao X. Cheng Z. Meng H.. (2013). Intercropping of green garlic (*Allium sativum* L.) induces nutrient concentration changes in the soil and plants in continuously cropped cucumber (*Cucumis sativus* L.) in a plastic tunnel. PLoS One 8:e62173. doi: 10.1371/journal.pone.0062173, PMID: 23637994 PMC3634817

[ref54] Xie K. X. Xue Z. H. Chen Z. D. (2021). Effects of intercropping different plants in tea garden on yield and quality of tea and soil of tea garden. J. Tea Commun. 48, 422–429. doi: 10.3969/j.issn.1009-525X.2021.03.007

[ref55] Yan F. Lou Y. H. Chen J. X. Zheng S. H. He W. Z. (2017). The effect of intercropping *Trifolium repens* on temperature humidity and growth of tea root system in tea plantation. Chin. J. Trop. Crops 38, 2243–2247. doi: 10.3969/j.issn.1000-2561.2017.12.008

[ref56] Yao Y. Zhang T. Ma W. W. Li L. Y. Li C. H. (2016). Effects of different intercropping patterns on photosynthetic physiology characteristics of tea plants and tea quality. J. Shanxi Agric. Sci. 44, 470–473. doi: 10.3969/j.issn.1002-2481.2016.04.12

[ref57] Yousuf B. Keshri J. Mishra A. Jha B. (2012). Application of targeted metagenomics to explore abundance and diversity of CO_2_-fixing bacterial community using cbbL gene from the rhizosphere of *Arachis hypogaea*. Gene 506, 18–24. doi: 10.1016/j.gene.2012.06.083, PMID: 22766402

[ref58] Zhang Y. S. Chen M. T. Guo J. Y. Zhang Y. Chen M. Guo J.. (2022). Study on dynamic changes of microbial community and lignocellulose transformation mechanism during green waste composting. Eng. Life Sci. 22, 376–390. doi: 10.1002/elsc.202100102, PMID: 35573133 PMC9077819

[ref59] Zhou Y. J. Li J. H. Ross Friedman C. Wang H. F. (2017). Variation of soil bacterial communities in a chronosequence of rubber tree (*Hevea brasiliensis*) plantations. Front. Plant Sci. 8:849. doi: 10.3389/fpls.2017.00849, PMID: 28611794 PMC5447074

[ref60] Zhu H. Y. Liu Z. D. Wang C. R. Zhu H. Liu Z. Wang C.. (2006). Effects of intercropping with persimmon on the rhizosphere environment of tea. Front. Biol. China 1, 407–410. doi: 10.1007/s11515-006-0054-3

